# Pre-Clinical In Vitro Models Used in Cancer Research: Results of a Worldwide Survey

**DOI:** 10.3390/cancers13236033

**Published:** 2021-11-30

**Authors:** Sarai Martinez-Pacheco, Lorraine O’Driscoll

**Affiliations:** 1 School of Pharmacy and Pharmaceutical Sciences, Panoz Institute, Trinity College Dublin, D02 PN40 Dublin, Ireland; smartin6@tcd.ie; 2 Trinity Biomedical Sciences Institute, Trinity College Dublin, D02 R590 Dublin, Ireland; 3 Trinity St. James’s Cancer Institute, Trinity College Dublin, D08 NHY1 Dublin, Ireland

**Keywords:** cancer, in vitro models, 3D culture, 3Rs, methodology, survey, precision medicine

## Abstract

**Simple Summary:**

Cancer cell lines, grown on plastic dishes i.e., two-dimensional (2D), are routinely used in cancer research, e.g., when evaluating the effectiveness of potential anti-cancer drugs before proceeding to studies in animal models and then human clinical trials. Stop/go decisions are generally made from these initial studies. As only ~10% of potential anti-cancer drugs succeed during clinical development, this suggests that these models are inadequate. Cells grown as three-dimensional (3D) models, akin to a tumor mass and with other cells that would naturally occur in its environment, should be more clinically relevant. We performed a worldwide survey, open to cancer researchers at all stages and in all settings, to find out what models they use; for what purposes, and why they chose those models. The majority reported using 2D models only, mainly due to lack of experience and costs but expressed interest in 3D cultures. Guidelines on how to develop such models cost-effectively are needed.

**Abstract:**

To develop and subsequently get cancer researchers to use organotypic three-dimensional (3D) models that can recapitulate the complexity of human in vivo tumors in an in vitro setting, it is important to establish what in vitro model(s) researchers are currently using and the reasons why. Thus, we developed a survey on this topic, obtained ethics approval, and circulated it throughout the world. The survey was completed by 101 researchers, across all career stages, in academia, clinical or industry settings. It included 40 questions, many with multiple options. Respondents reported on their field of cancer research; type of cancers studied; use of two-dimensional (2D)/monolayer, 2.5D and/or 3D cultures; if using co-cultures, the cell types(s) they co-culture; if using 3D cultures, whether these involve culturing the cells in a particular way to generate spheroids, or if they use additional supports/scaffolds; techniques used to analyze the 2D/2.5D/3D; and their downstream applications. Most researchers (>66%) only use 2D cultures, mainly due to lack of experience and costs. Despite most cancer researchers currently not using the 3D format, >80% recognize their importance and would like to progress to using 3D models. This suggests an urgent need to standardize reliable, robust, reproducible methods for establishing cost-effective 3D cell culture models and their subsequent characterization.

## 1. Introduction

Cancer research requires in vitro models capable to produce reliable biomedical information through mimicking the cells’ phenotype as it exists in the target tissue [1]. Thus, the use of pre-clinical in vitro models, as well as in vivo models, continues to be crucial in cancer research. These models are necessary for deciphering molecular mechanisms of key events such as tumor growth, metastasis, drug resistance, and aspects of immune evasion. They are also necessary for anti-cancer drug screening and development [2]. However, as only 10% of potential anti-cancer drugs succeed during their clinical development, mainly due to a lack of efficacy or intolerable toxicity [3,4,5], this puts into question the relevance of the models used.

Before any cancer research progresses to clinical utility, it typically involves studies in animal models. However, for many reasons, including ethics and the 3Rs (replacement, reduction, and refinement of the inclusion of animals in research), costs, complicated and laborious techniques requiring research specialists, appropriate in vitro models should always be used first to their maximum potential [6,7].

The simplest approach for in vitro cancer studies is the monolayer culture of cancer cells in two-dimensional (2D) conditions. A 2D culture is straightforward with and low-cost maintenance, which might be considered one of their main advantages [8].

Nevertheless, its limitations have been increasingly recognized, proven its inadequacy as a fully reliable pre-clinical tumor model, mainly as an over-simplified version of tumor conditions in vivo, often failing to address many of the more dominant pathological problems, such as the tumor microenvironment (TME). Moreover, it is reported that 2D cultures do not conserve the original shape and polarization of cells [7,9], which could affect other properties such as their functions, organelles’ organization and cell signaling [8].

From the 1980s when Mina Bissell highlighted the importance of the extracellular matrix (ECM) in cell behavior [10,11,12,13], it is generally accepted that three-dimensional (3D) cell culture models (if developed appropriately) should more accurately represent the tumor and its microenvironment and that their behavior should be more reflective of in vivo cellular responses when compared to the 2D models [1,14,15,16].

To make real advances in developing precision medicine for cancer, pre-clinical models that represent in vivo biology and the microenvironmental factors, while also respecting the rights of animals, are necessary [2]. Indeed, several approaches are being used to increase the complexity of the models. These include 2D co-cultures of, e.g., cancer cells with stromal cells; 2.5D cultures, which consist of cells growing on top of a layer of extracellular matrix (ECM) proteins; and 3D cultures which are more complex structures [15,17,18]. Three-dimensional cultures can be designed using different approaches and divided into: (i) non-scaffold-based cultures or spheroids; (ii) scaffold-based cultures; (iii) specialized 3D culture platforms, including microfluidic cell culture platforms and organ-on-chip systems that allows the control of different conditions (e.g., the creation of chemical gradients by the fluid flow) [19], and (iv) hybrid systems that integrate spheroids into a scaffold structure [20], providing improved tumor models for screening anti-cancer drugs.

**Figure 1:**
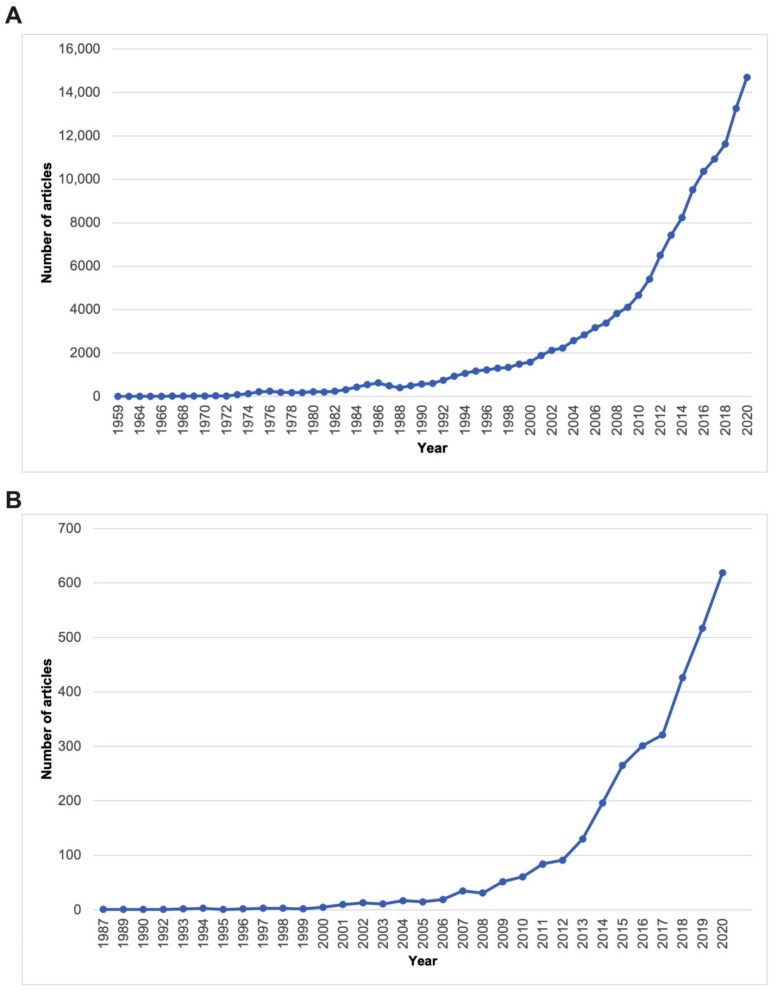
Expansion in the numbers of peer-reviewed published articles with the term “in vitro tumor models” *(***A**) or “3D in vitro tumor models (**B**) (source: Scopus; Accessed over time, most recently 17 November 2021).

Using the bibliometric tool Scopus to identify growth metrics of the terms “in vitro tumor models” and “3D in vitro tumor models”, a substantial increase in publication numbers was observed (Figure 1), year on year, indicating increasing interest in this field.

Despite the general agreement in the cancer research community that in vitro 3D models can be more representative of tumors in the body than 2D models, these approaches have not been extensively incorporated in research [9,21].

Reviewing the published literature gives us information on what in vitro models were used in research that was published. However, it gives us no indication on why such models were chosen—or, indeed, what models are being used in unpublished cancer research and why. Thus, this study aimed to perform the first global survey of currently used pre-clinical in vitro models for cancer research, the reasons for the choices made, and the considered strengths and limitations of these models.

## 2. Methods

### 2.1. Survey Design

Survey questions (n = 40) were generated by using Typeform^®^ (Barcelona, Spain; http://typeform.com) and were designed with logic jumps, meaning that respondents were only brought to certain sections based on their previous answers. The survey design is shown in the Supplementary Information (Supplementary Material S1). After obtaining ethics approval including GDPR considerations from the School of Pharmacy and Pharmaceutical Sciences Research Ethics Committee of Trinity College Dublin (No. 2020-04-01), the survey was circulated extensively to cancer research centers by email and by sharing on social media channels. The survey was opened on 28 May 2020 and closed in December 2020. A total of 101 full submissions were collected to an Excel file and consequently analyzed.

### 2.2. Statistical Analysis

Analysis was performed using IBM SPSS 28. Nominal data are presented as percentages. The existence of correlation between variables was assessed using the Pearson correlation coefficient (PCC).

## 3. Results

**Figure 2:**
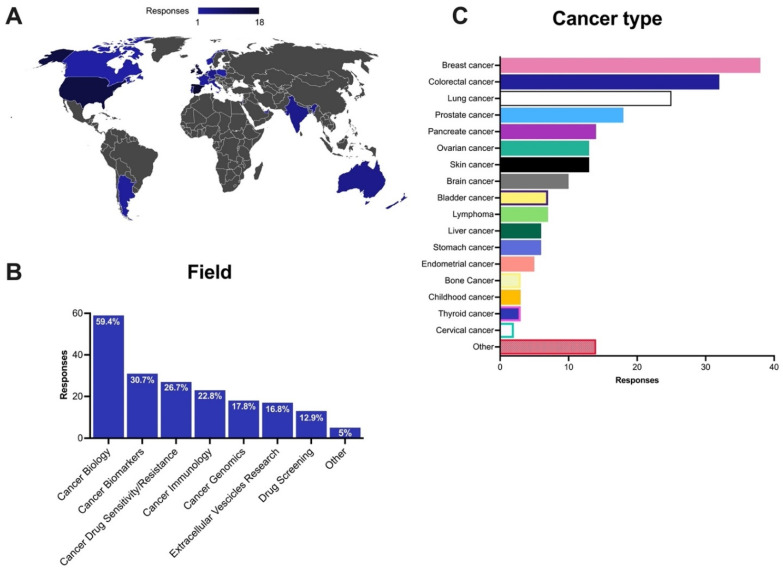
General profile of the respondents. (**A**) Number of respondents categorized by the country of work. The graph was obtained from 101 responses; (**B**) Field of cancer research performed; (**C**) Cancer type studied by the respondents. Note: some respondents indicated that they are involved in more than one field of research and/or work on more than one cancer type.

The extensive reach of the survey was clear by the fact that the respondents were from 19 countries spanning four continents (Figure 2A). Any relatively strong correlations (PCC > 0.4) or strong negative correlations (PCC < −0.4) were found between continent and cancer type or field. Of the respondents, 96% belonged to academia, 3% from clinical settings, and 1% from industry. Principal investigators (39.6%), post-doctoral researchers (17.8%) and senior researchers (8.9%) represented more than 66% of the responses, reflecting a strong level of interest in the topic at the senior level. Respondents also included PhD students (26.7%), clinical researchers (2%), as well as associate researchers (1%), laboratory technicians (1%) and research assistants (1%).

Regarding the fields of cancer research performed by the respondents, the most reported was fundamental cancer biology, followed by cancer biomarkers and cancer drug sensitivity/resistance (Figure 2B). No relatively strong correlations (Pearson’s R > 0.4) nor strongly negative correlations (PCC < −0.4) were found between field and cancer type studied. Interestingly for a relatively new and specialized field of research, extracellular vesicles research in cancer was reported as the interest of almost 17% of respondents. The cancer types represented by the in vitro models varied widely. The most common cancer types being studied were breast, colorectal, lung and prostate cancer. Cervical cancers were the most under-studied based on the responses (see Figure 2C). 

### 3.1. Principal Characteristics of In Vitro Models

A key component of any in vitro tumor model is the source of cancer cells. Commercially available cell lines were the most widely chosen option, accounting for 81.2% of the responses; with primary cells accounting for 38.6%, and other undeclared sources making up the remaining approximately 5%.

The vast majority of the respondents indicated that cancer cells are the principal cell type for their in vitro models (91.1%) whilst the second most used were immune cells (20.8%), followed by stem cells (7.9%) and stromal cells (6.9%). Of those who reported working with cancer cells, 93.8% indicate that these were of primary tumor origin; 21.9% reported that their studies involved working with cells from the pre-metastatic niche; 25%, from the non-tumor cell part of the metastatic niche; and only 18.8% were secondary tumor cells.

**Figure 3:**
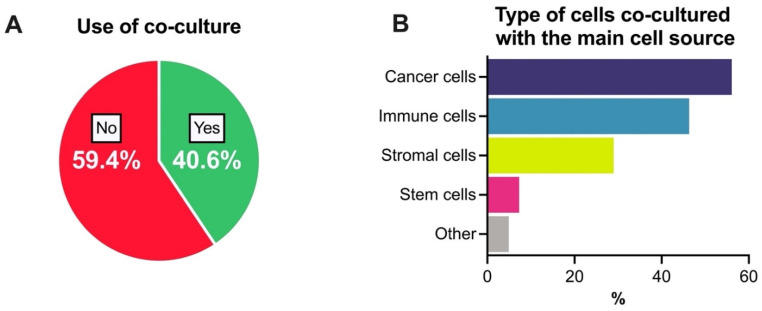
Co-culture use in pre-clinical in vitro tumor models. (**A**) Use of co-culture models and (**B**) types of cells used in these co-culture models together with the main cell source. Results are represented as percentages (%). Note: Figure 3B represents a question with multiple choice. Some respondents indicate that their co-culture models involved more than one type of cell.

Co-culture is an approach in which two or more different types of cells (rather than just cancer cells alone) are cultured together to better represent a tumor and the tumor microenvironment (TME) [22]. However, only 40.6% of the researchers reported that they use any co-culture model, in any form of in vitro model (Figure 3A). When we asked what type of cells were co-cultured with their main cell source, the researchers indicated cancer cells (56.1%), immune cells (46.3%), stromal cells (39%) and stem cells (7.3%) (see Figure 3B).

### 3.2. Three-Dimensional Models and Types Used in Cancer Research

**Figure 4:**
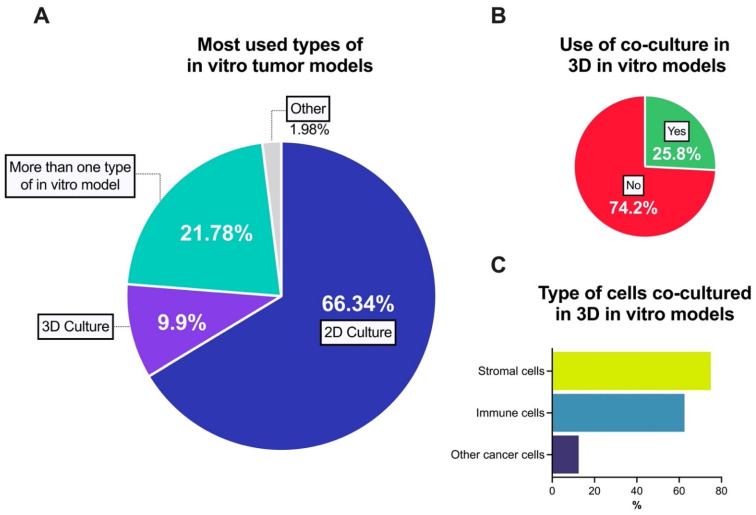
Use of 3D in vitro models in pre-clinical cancer research. (**A**) Main in vitro tumor models; (**B**) Use of co-cultured approach related to 3D in vitro tumor models; (**C**) Most used cell types to co-culture with cancer cells in 3D in vitro tumor models. Note: Figure 4C represents a question with multiple choice. Some respondents indicate that their co-culture models involved more than one type of cell.

The development of in vitro tumor models with increased complexity has been aided by our improved understanding of tumor biology, tissue engineering, as well as advancements in the development of biomaterials and microfluidics [2]. However, despite such progress the use of 2D culture models exclusively is still being the most preferred option; followed the use of more than one in vitro model but using of 2D culture in all cases, and the use of 3D culture exclusively was the third chosen option (Figure 4A).

When the correlations between the cell types used and the culture types were analyzed, it was found relative strongly negative correlations between the use of stem cells in 2D Culture (PCC = −0.459; *p* = <0.001) and the use of cells obtained from the metastatic niche in 3D Culture (PCC = −0.419; *p* = <0.001). Regarding the positive correlations, a relative strongly correlation was found between the use of primary cells in 3D Culture (PCC = 0.442; *p* = <0.001).

We also asked about the use of co-culture when using 3D models specifically. Fewer than 26% of studies used co-cultures, while 74.2% of 3D constructs were monocultures (Figure 4B). In the cases was used, stromal cells (75%) and immune cells (62.5%) were the most used type of cells in combination with cancer cells. Only 12.5% of respondents indicated the use of other cancer cells in a co-culture (Figure 4C).

**Figure 5:**
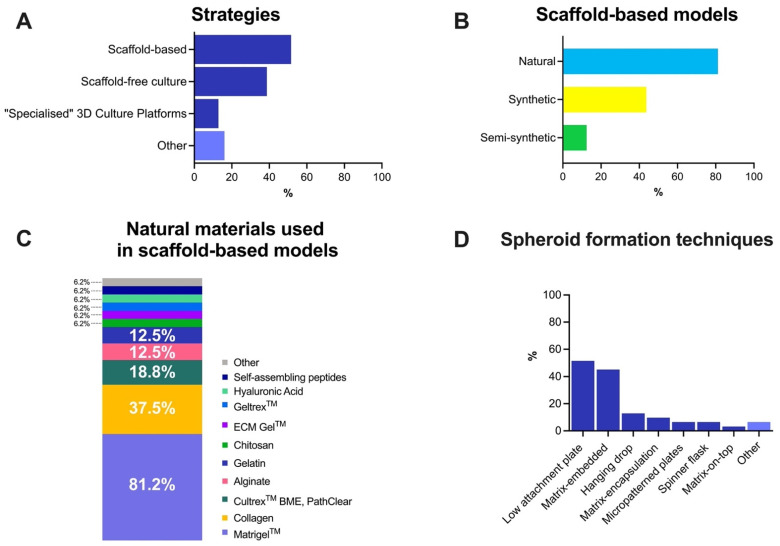
Overview of 3D culture models in cancer research. (**A**) Main strategies used for 3D models; (**B**) Types of scaffold-based models used in cancer research; (**C**) The most used natural materials in scaffold-based models; (**D**) Procedures used for spheroid formation. Note: some respondents indicated that they used more than one strategy, scaffold-based model type and/or spheroid formation technique. Results are represented as percentages (%).

The survey also included questions related to the characteristics of the principal 3D model used (see Figure 5). For those using 3D models at all, it was found that the most extended approach used was scaffold-based models (51.6%), followed by scaffold-free culture (38.7%) (Figure 5A). No one reported using a hybrid system. A natural scaffold was the preferred option for those who used scaffold-based models (81.25% of the respondents), followed by synthetic (43.8%), and then semi-synthetic (12.5%) materials (Figure 5B). A list of materials that might be used was included in the questions for the three categories (see Supplementary Material S1). Only two synthetics (Polyethylene glycol, PEG (31.2%); Polyhydroxy ethyl methacrylate, p-HEMA (6.2%)) and two semi-synthetic materials (PEGylated protein scaffolds, 23.1%; HyStem^TM^, 7.7%) were reported to be used by any of the respondents. Natural materials were widely used (Figure 5C). We also queried the functionalization of the materials used. Most (62.5%) of the respondents indicated that the material that they used in their 3D models was not functionalized, while 25% were (the remaining 12.5% indicated that the functionalization was not applicable).

Another approach used in 3D culture is multicellular tumor spheroids, dense tri-dimensional aggregates of cancer cells alone or combined with other cell types in suspension culture and where a scaffold is not used [6,23]. Various techniques can be used to produce such spheroids. When we asked about the procedures used for spheroid formation, researchers indicated that the main three approaches used were low attachment plate (51.6%), matrix-embedded (45.2%) and hanging drop (12.9%), among others (Figure 5D).

Regarding the number of different 3D model types used by any given researcher, 64.5% of respondents using 3D culture indicate use just one model, whilst the remaining 35.5% use more than one 3D culture. The characteristics of these additional 3D models are collected in Supplementary Figure S1.

### 3.3. Characterization of the Models and Downstream Applications

**Figure 6:**
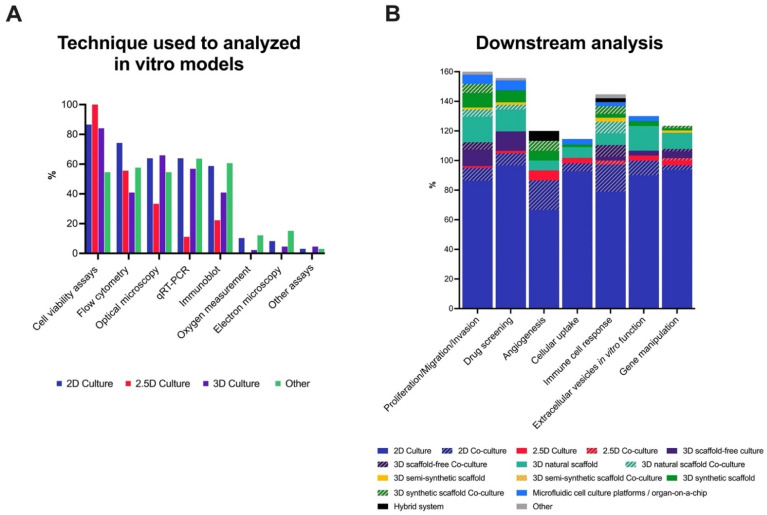
Analysis of pre-clinical in vitro model and their main applications. (**A**) Most used techniques to analyze in vitro models.; (**B**) Choice of cell culture models for application in the various downstream applications. Most commonly used in vitro models used for proliferation/migration/invasion assays, drug screening analysis, angiogenesis assay, cellular uptake/release assays, immune cell response assays, extracellular vesicles in vitro functional assay and gene manipulation assays. Bars represent the percentage for each option, with respondents being able to choose more than one option as appropriate. Percentages were calculated based on the reported use of each technique.

Analyzing the techniques used to characterize the cells of the different types of models, we observed that cell viability assays were the most commonly used method to analyze the cells in 2D, 2.5D and 3D culture. This was followed, in popularity, by flow cytometry and optical microscopy. Another trend we can observe in the case of other models, where the most used techniques were quantitative reverse transcription PCR (RT-qPCR), immunoblotting and flow cytometry (see Figure 6A).

The intended downstream application may influence the choice of cell culture models for cancer research. Here, we asked researchers what model(s) they used for some of the most common assays used in cancer research: i.e., proliferation/migration/invasion assays, drug screening assays, angiogenesis assay, cellular uptake/release assays, immune cell response assays, extracellular vesicles (EVs) in vitro function assays, and gene manipulation. Unsurprisingly, 2D culture was the most used method for all the downstream applications (see Figure 6B).

Correlations between the techniques used to analyze the different in vitro models and the downstream analysis were evaluated and those PCC values above 0.4 (considered relatively strong) were summarized in Table 1. No strongly negative correlations were found between the use of any technique to analyze in vitro models and the downstream analysis.

## 4. Benefits and Limitations of In Vitro Models in Cancer Research

Although the interest in and the use of 3D models has increased over the last decade, these models are still used by the minority of cancer researchers who perform in vitro studies.

**Figure 7:**
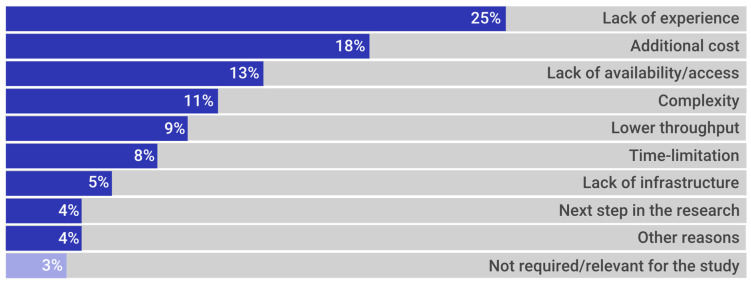
Principal reasons for not using 3D models (%).

We asked those who indicated not using 3D culture models at all about the main reasons why this was so. The principal reasons given were lack of experience (25%) and the additional cost when compared to 2D cultures (18%). However, 4% of the respondents indicated an intent to develop a 3D culture in the next step of their research (Figure 7).

The time required for the establishment of model de novo was reported to be on average 6 weeks. It was also asked about the time needed not only to establish a model de novo but also the time required to routinely apply the model. Similarly, the time required to be able to use a model was reported as, on average, 6 weeks. Obviously, the time required for the development depends on the complexity of the model and would be a principal reason why the times could vary between different models.

Researchers were also asked to give their opinion on the benefits and limitations of using 3D models. In relation to the advantages, as predicted, some respondents reported that these models are more realistic and better represent the tumor physiopathology and microenvironment when compared with 2D models. Other answers included: better-represented tumor growth, better-replicated metabolism, or considering the cell–cell interactions. Three-dimensional models were considered less time-consuming and less expensive than in vivo models and the importance of reducing the use of animals was highlighted. When researchers were asked about their interest in using 3D culture models, 81.3% expressed interest, 11.9% remained neutral, and only 7% of the respondents indicated no/low interest. However, only 33.7% agreed with the proposal that 2D cell culture models should be completely replaced by 3D cell culture models, 23.8% remained neutral, and 42.7% disagreed with this suggestion.

Regarding limitations, respondents predominantly reported the limitations as lack of reproducibility and reliability, combined with variability in results, due, at least in part, to an absence of standardized protocols for establishing 3D models. These issues include those with assays developed for 2D systems that are not directly translatable to 3D models. In fact, approximately 57% of those surveyed indicated their main concern with the utilization of 3D culture models is that many routine assays used with 2D cultures are not translatable to 3D. It is noteworthy that only 28.7% of the respondents indicated that they have developed and published a protocol for a novel in vitro model that they developed themselves. Issues were also raised about the necessity of additional expertise, time, and consumables required to develop, maintain, and optimize 3D models compared to classic 2D models. The point was also made that, in some circumstances, 3D models have no additional benefits compared to 2D models; for example, when trying to mimic the immune response in cancer. So, adding this additional step between 2D and necessary pre-clinical in vivo studies was considered by some as not relevant to their research.

Considering the use of in vivo models, 56.4% of the respondents reported that—in addition to in vitro models—they also use in vivo models in their laboratory, but only 40.4% of those based on their research predominantly perform in vivo studies. The principal reasons to do not use in vivo models were lack of availability (30%), lack of resources (26.7%) and the 3Rs (18.3%). Only 11.7% of the researchers simply indicate that the choice to not include in vivo models was a personal choice. Most of the respondents agreed that it is still not feasible to completely replace in vivo animal models with 2D/2.5D/3D in vitro models (83.3%). However, if both in vitro models and in vivo models could achieve the same benefits for cancer research, they would prefer to use an in vitro approach.

Concerning the advantages and limitations of this survey, an advantage of the approach used for this worldwide survey includes the fact that it was open to anyone in the world who wished to partake. The survey was widely advertised via social media and via email to reach as many interested parties as possible. The fact that time and appropriate steps were invested in getting ethics approval to complete the survey and to ensure that no personal data were collected meant that respondents could be confident of the integrity of the research; that they could answer completely honestly with no concern that their name would be associated with any response; and that their contact details would not be used for any other purpose. Another advantage was the fact that it was not mandatory for respondents to answer all questions if they wished to avoid some, and they could complete as much of the survey as they wished. The design of the survey (logic jumps) meant that respondents were only brought to certain sections, based on their previous answers. This meant that respondents did not have to spend time reading through questions that were not relevant to them, based on their answer to a previously presented question. The main limitation of the survey—as with any such survey—is that there are people working with preclinical in vitro models in cancer research who the survey may not have reached or who were not interested in completing the survey. Thus, the responses achieved are, of course, only from those who were interested in taking the time to complete the survey.

## 5. Conclusions and Final Remarks

Although 3D in vitro models are increasingly used and there are increasing numbers of support structure and/or co-culture options for this, the use of 2D models only is still most typical among cancer researchers. It is generally accepted that, although 2.5D and 3D cultures/co-cultures are a stepping-stone between 2D cultures and animal models when trying to answer many questions in cancer research, they are more expensive, more time-consuming, and require a higher level of expertise; and yet none of them is complex and sophisticated enough to substitute animal models. Therefore, although the use of 2D/2.5D/3D) pre-clinical in vitro models can reduce the need for animals in some areas of cancer research, they still cannot mimic in full the heterogeneity of tumors and their microenvironment as they exist in vivo. The inclusion of organoids offers a lot of promise Furthermore, the inclusion of mathematical models/in silico procedures could help to reduce wet-laboratory experiments, complementing in vitro models [24] and helping to advance to achieving personalized medicine [25].

However, the relatively limited availability of these to many academic and industry cancer researchers means that other options will continue to be needed. Concerted efforts are now needed to bring academia and industry together to develop the most sophisticated 3D models possible, to develop guidelines and standard operating procedures for the establishment of the same in the interest of rigor, reproducibility, standardization, and best practice. These need to be as cost-effective as possible. In parallel, rather than trying to apply downstream analyses optimized for 2D models, there is an urgent need to develop compatible downstream analyses for 3D cancer models.

As things stand in cancer research, animal models are still needed following all currently available in vitro models. However, in vivo approaches have inherent limitations including the prohibitive costs in large animal studies and differences in cancer development between species and ethical concerns [26].

Two-dimensional in vitro models were shown not to fully represent the architecture, heterogeneity and complexity of human tumors and more representative models are needed to better reflect key aspects of tumor biology. Thus, there is an urgent need for standardized methods and the establishment of guidelines [27] and/or a transparent knowledge base for the development and characterization of more sophisticated 3D cell culture/co-culture models. Additionally, these improvements should be beneficial in pre-clinical studies, advancing on current approaches and even substituting any current methods that are not ideal. This clearly indicates that additional research in this field is needed to improve preclinical in vitro models that replicate the key elements of tumor complexity and heterogeneity and find reliable alternative strategies to animal models.

## Figures and Tables

**Table 1:** Significant correlations between techniques used to analyze in vitro models and downstream analysis (PCC > 0.4).

Type of In Vitro Model	Technique to Analyze the Model	Downstream Analysis	PCC	*p*-Value
2D models	Cell viability assays	Proliferation	0.448	<0.001 (**)
	qRT-PCR	Gene manipulation	0.412	<0.001 (**)
2.5D models	Flow Cytometry	Immune cell response (2.5D Co-culture model)	0.492	<0.001 (**)
	Optical Microscopy	Immune cell response (2.5D Co-culture)	0.704	<0.001 (**)
	Flow Cytometry	Gene manipulation (2.5D Co-culture model)	0.492	<0.001 (**)
	Optical Microscopy	Gene manipulation (2.5D Co-culture model)	0.704	<0.001 (**)
3D models	Immunoblot	Proliferation (Scaffold-free model)	0.49	<0.001 (**)
	Flow cytometry	Proliferation (Natural scaffold-based model)	0.459	<0.001 (**)
	Optical microscopy	Proliferation (Natural scaffold-based model)	0.489	<0.001 (**)
	qRT-PCR	Proliferation (Natural scaffold-based model)	0.411	<0.001 (**)
	Immunoblot	Proliferation (Natural scaffold-based model)	0.439	<0.001 (**)
	Flow cytometry	Drug screening (Natural scaffold-based model)	0.417	<0.001 (**)
	Optical microscopy	Drug screening (Natural scaffold-based model)	0.505	<0.001 (**)
	Cryosectioning	Drug screening (Natural scaffold-based co-culture model)	0.454	<0.001 (**)
	Electron microscopy	Drug screening (Natural scaffold-based co-culture model)	0.49	<0.001 (**)
	Electron microscopy	Gene manipulation (Scaffold-free co-culture model)	0.704	<0.001 (**)
	Flow cytometry	Gene manipulation (Natural scaffold-based model)	0.502	<0.001 (**)
	Other	Angiogenesis (Synthetic scaffold-based model)	0.704	<0.001 (**)
Other models	Oxygen measurement	Immune cell response	0.492	<0.001 (**)

**. Correlation is significant at the 0.01 level (two-tailed).

## Data Availability

The data presented in this study are available on request from the corresponding author.

## Supplementary Materials

The following are available online at https://www.mdpi.com/article/10.3390/cancers13236033/s1, Figure S1: Types of additional 3D models most used by respondents, Material S1: Survey Questionnaire.
