# Supplementary material for: Pre-Clinical In Vitro Models Used in Cancer Research: Results of a Worldwide Survey
Source: Cancers (Basel). 2021 Nov 30;13(23):6033. doi: 10.3390/cancers13236033 (PMC8656628; doi:10.3390/cancers13236033)
Supplement: Supplementary file 1 [file cancers-13-06033-s001.zip › cancers-1335376-supplementary.pdf]

Supplementary Material

Pre-clinical in vitro models used in cancer research: results of a worldwide survey

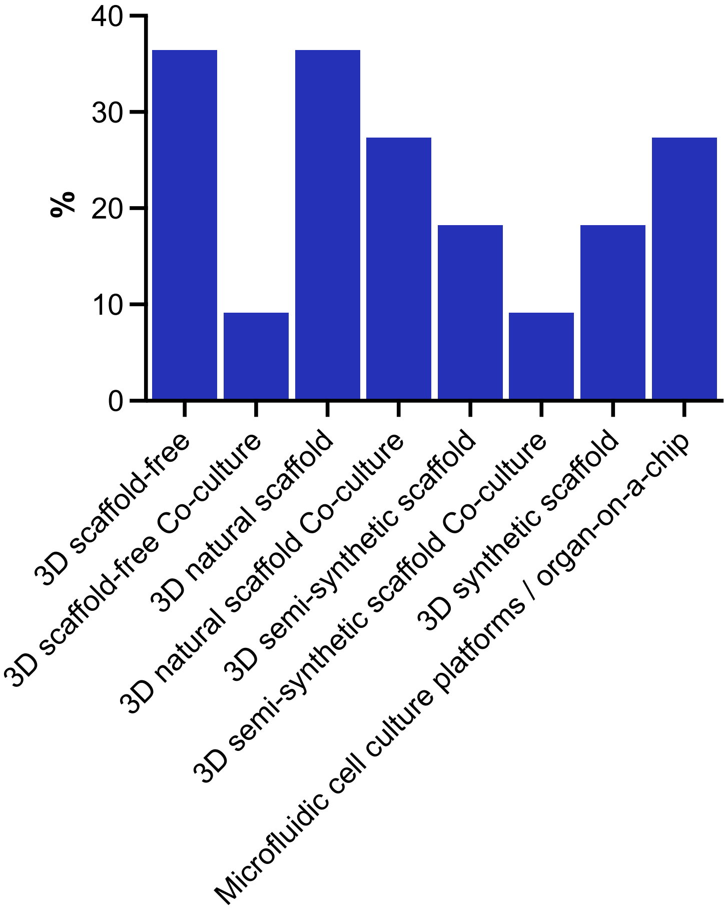

Figure S1. Types of additional 3D models most used by respondents (%). Note: some respondents indicated to use more than one additional 3D models.

Material S1—Survey Questionnaire

Pre-clinical in vitro models used in cancer research: results of a worldwide survey

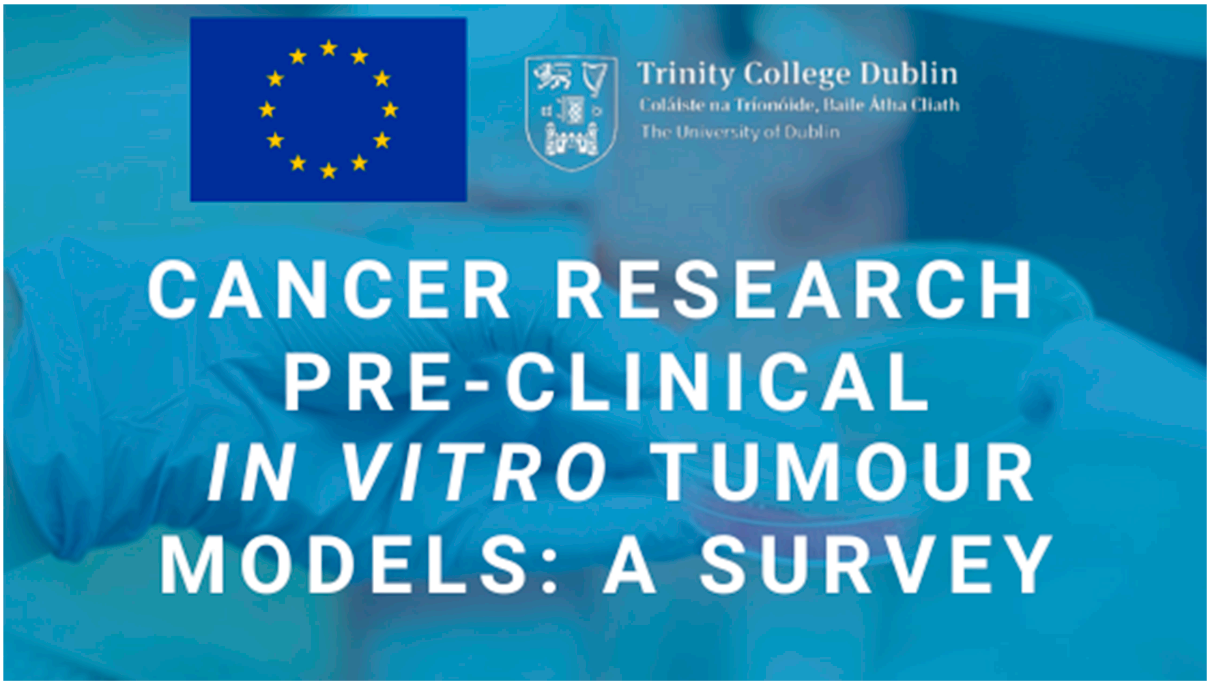

Participant Information and Consent

Substantial efforts have been made over recent years to develop *in vitro* tumor models with increased complexity to better represent tumors in the body and to reduce animal testing. However, there are still challenges in developing realistic *in vitro* models that reproduce the complexity of tumor tissue and stromal architecture, as it exists in the body. And, although several types of 3D culture models are being developed, monolayer (2D) cell-based assays remain an important pillar in cancer research. The goal of our research is to collate information on the uses of pre-clinical *in vitro* models and the reasons for the choices made.

We invite individuals, who are 18 years or older and who have knowledge working with pre-clinical cancer research models, to please complete this survey, which should take only 15 minutes of your time. Participation in this research survey is voluntary. This survey is anonymous, so if you decide to take part, responses cannot be withdrawn after submission, as respondents cannot be traced.

The Survey was designed by me, Sarai Martinez Pacheco (a H2020-MSCA-TRAIN-EV PhD student working with Prof. Lorraine O'Driscoll at Trinity College Dublin, Ireland). When the Survey is completed, the results will be analyzed and disseminated so that we can all learn from this.

This study has been approved by the School of Pharmacy & Pharmaceutical Sciences (SoPPS) Level 1 Research Ethics Committee (REC), Trinity College Dublin on 25 May 2019.

If you have any questions about the survey or need technical support, please contact at me smartin6@tcd.ie

We really appreciate your input!

Survey questions (* mandatory questions)

1. By submitting this form, I am indicating that: *

- I am 18 years of age or older.

- I have knowledge of working with pre-clinical cancer research models.
- I have read and understand the participant information above and voluntarily agree to participate in this research study.

  - I consent → **Start Survey**
  - I do not consent → **End**

2. In which **country** do you work? *
   - Dropdown Question
   - Options: List of countries

3. Optional: Please provide the name of the **institution** where your work is located
   - Comment box

4. Select your **sector***
   - Checkboxes question
   - Options:
     - Academia
     - Industry
     - Clinic

5. Which of the following best describes your **career stage/ institution role**? *
   - Dropdown question
   - Options:
     - Undergraduate Researcher
     - MSc Student
     - PhD Student
     - Research Assistant
     - Clinical Researcher
     - Laboratory Technician
     - Post-doctoral Researcher
     - Associate Researcher
     - Senior Researcher
     - Principal Investigator
     - Other (specify)

6. Which of the following best describes your **field** of **cancer research**? *
   - Multiple Choice question
   - Options:
     - Cancer Biology
     - Cancer Genomics
     - Cancer Biomarkers
     - Drug Screening
     - Cancer Drug Sensitivity/Resistance
     - Cancer Immunology

- Extracellular Vesicles Research
- Other (specify)

7. What **cancer type(s)** are you studying? *

- Multiple choice question
- Options:
  - Lung cancer
  - Brain cancer
  - Skin cancer
  - Liver cancer
  - Bone cancer
  - Colorectal cancer
  - Lymphoma
  - Breast cancer
  - Bladder cancer
  - Stomach cancer
  - Pancreatic cancer
  - Prostate cancer
  - Childhood cancer
  - Endometrial cancer
  - Uterine cervical cancer
  - Thyroid cancer
  - Ovarian cancer
  - Other (please specify)

8. What is your **main cell source**? *

- Multiple choice question
- Options:
  - Primary cells
  - Commercially available cell lines
  - Other (specify)

9. What type(s) of cells is your **main cell source**? *

- Multiple choice
- Options:
  - Cancer cells
  - Stem cells
  - Immune cells
  - Stromal cells
  - Other: (specify)

If the answer in Q8 is Primary cells and the answer in Q9 is cancer cells → **Go to Q10**
All other cases → **jump to Q11.**

10. Which of the following characteristics best describe your cancer cells? *

- Multiple choice
- Options:
  - Primary tumor cells
  - Secondary tumor cells
  - Cells obtained from pre-metastatic niche
  - Cells obtained from metastatic niche
  - Other (specify)

11. Do you use **co-culture** model(s)? *

- Yes ➝ **Q12**
- No ➝ **Q13**

12. What type(s) of **cells** do you **co-culture** with your main cell source? *

- Multiple choice
- Options:
  - Cancer cells
  - Stem cells
  - Immune cells
  - Stromal cells
  - Other: (specify)

13. What **type(s)** of *in vitro* tumor model(s) do you use **mostly**? *

- Checkboxes question (at least 1)
- Options:
  - 2D Culture
  - 2.5D Culture
  - 3D Culture
  - Other (specify)

If the answer is:

If the answer is:	Go to:
2D	**Q28**
2.5D	**Q27**
3D	**Q14**
Other	**Q28**
2D+2.5D	**Q27**
2D+3D	**Q14**
2D+Other	**Q28**
2.5D+3D	**Q14-Q27-Q29**

2.5D+Other	**Q27**
3D+Other	**Q14**
2D+2.5+3D	**Q14-Q27-Q29**
2D+2.5D+Other	**Q27-Q29**
2D+3D+Other	**Q14-Q29**
2.5+3D+other	**Q14-Q27-Q29**
2D+2.5D+3D+Other	**Q14-Q27-29**

14. The purpose here is to collect the **main characteristics** of the 3D culture that you use **the most**. If you use more than one, indicate it later in the survey

- Multiple choice question
- Options:
  - (A) Scaffold-based
  - (B) scaffold-free culture
  - (C) "Specialized" 3D Culture Platforms (Microfluidic cell culture platforms / organ-on-a-chip)
  - (D) Hybrid System
  - (E) Other (specify)

If the answer contains (A) → **Q15**

If not → **Q16**

15. Scaffold-based related questions

a. Which of the following types of **scaffold-based models** do you use to develop your model?

- Multiple choice question
- Options:
  - Natural Scaffold
  - Synthetic Scaffold
  - Semi-synthetic Scaffold (biohybrid)
  - Other (specify)
  - Not applicable

b. Which of the following **natural materials** do you use in your principal 3D model?

- Multiple choice question
- Options:
  - Not applicable
  - Matrigel™
  - Cultrex™ BME, PathClear
  - ECM Gel™
  - ECL Cell Attachment Matrix™
  - Geltrex™
  - HuBiogel™

- Collagen
- Gelatin
- Fibronectin
- Fibrin
- Glucan
- Hyalouronic Acid
- Chitosan
- Alginate
- Agarose
- Self-assembling peptides
- Nanofibrillar cellulose scaffolds (NFC)
- Other (specify)

c. Which of the following **synthetic materials** do you use in your principal 3D model?

- Multiple choice question
- Options:
  - Not applicable
  - Polyethylene glycol (PEG)
  - Poly(ethylene oxide) (PEO)
  - Poly(acrylic acid) (PAA)
  - Poly(vinyl alcohol) (PVA)
  - Polyglycolic acid (PGA)
  - Poly-L-lactic acid (PLA)
  - Poly(hydroxyethyl methacrylate) (p-HEMA)
  - Poly(methacrylic acid) (PMMA)
  - TrueGel3D™
  - Other (specify)

d. Which of the following **semi-synthetic materials** you use?

- Multiple choice question
- Options:
  - Not applicable
  - PEGylated protein scaffolds
  - Gelatin methacrylamide (GelMA)-based scaffolds
  - HyStem™
  - Other (specify)

e. Is your scaffold **functionalized**?

- Options:
  - Yes → **Go to Q15f**
  - No → **Go to Q16**
  - Not applicable → **Go to Q16**

f. If yes, please give more **details** on the **functionalization**:

- Comment

16. What technique do you use for the **spheroid formation**?
- **Multiple choice question**
- Options:
  - Not applicable
  - Hanging drop
  - Low attachment plate
  - Magnetic levitation
  - Pellet culture
  - Microgravity bioreactors
  - 3D Bio-printing
  - Matrix-on-top
  - Matrix-embedded
  - Matrix-encapsulation
  - Spinner flask
  - Micropatterned plates
  - Other (specify)

If the answer in Q14 was:

If the answer was	Go to
Hybrid system	Q17
Not Hybrid system	Q18
Not Hybrid system, not other, but was Specialized platform	Q19
All other cases	Q20

17. If you select Hybrid System, please briefly **describe** the hybrid system used:
- Comment

If the Q14 was Other → **Q18**
If the Q14 was not Other and was Specialized platform → **Q19**
All other cases jump → **Q20**

18. If you select Other, please briefly **describe** the system used:
- Comment

If the Q14 was Specialized platform → **Q19**
All other cases jump → **Q20**

19. "Specialized" 3D Culture platforms related questions

a. Which of the following **"Specialized" 3D Culture platforms** do you use?
- Multiple choice question

- Options:
  - CellASIC® ONIX Microfluidic Plates
  - Quasi Vivo®
  - Organovo (ONVO)
  - 2-OC and 4-OC (TissUse)
  - OrganoPlate®
  - Other (specify)

20. Do you use **co-culture** in your main **3D model**?
    - Yes → **Go to Q23**
    - No → **Go to Q24**

21. What type of **cells** do you culture **in combination** with your cancer cells **in this model**?
    - Options:
      - Other cancer cells
      - Stem cells
      - Immune cells
      - Stromal cells
      - Other: (specify)

22. How many **weeks** are required for successful establishment of model *de novo*?
    - Comment (Insert number)

23. How many **weeks** are required before using the model for a set of experiments?
    - Comment (Insert number)

Additional 3D Models

If you develop/use more than one different 3D culture model, we would appreciate if you could complete this part of the survey too

24. Do you use more than one 3D Culture model?
    - Options:
      - Yes. → **Go to question 25**
      - No → **Go to question 26**

25. Please choose which of the following options best describe your additional 3D models (Tick all that apply)
    - Multiple choice question
    - Options:
      - 3D natural scaffold
      - 3D natural scaffold Co-Culture
      - 3D synthetic scaffold
      - 3D synthetic scaffold Co-Culture
      - 3D semi-synthetic scaffold
      - 3D semi-synthetic scaffold Co-Culture

- 3D scaffold-free culture
- 3D scaffold-free culture Co-Culture
- Microfluidic cell culture platforms / organ-on-a-chip
- Hybrid System
- Other (specify)

26. Please use this space for additional comments related to 3D-culture (commercial kits used to develop the models, biochemical signals, etc.) **(not mandatory)**
    - Comment

27. Which of the following strategies do you use for your **2.5D Culture**?
    - Multiple choice question
    - Options:
        - Microwells
        - Topographic patterning
        - Other (specify)

28. What are the **main reasons** why you **do not use** 3D Culture models?
    - Multiple choice question
    - Options:
        - Lack of availability/access
        - Lack of infrastructure
        - Additional cost
        - Lack of experience in appropriate skill
        - Lower throughput
        - Complex, difficult-to-replicate systems
        - Time-limitation
        - Other: (specify)

29. What technique(s) do you **use** for the **analysis of the cells** in the model(s)? *

Please, select the technique(s) carried out for each type of *in vitro* model

a. What technique(s) do you **use** for the **analysis of the cells** in the **2D model(s)**?
    - Multiple choice
    - Options:
        - I do not use 2D models
        - Cell viability assays
        - Optical microscopy
        - Electron microscopy
        - Flow cytometry
        - Immunoblot
        - qRT-PCR
        - Cryosectioning

- Oxygen measurement
- Other (specify)

b. What technique(s) do you **use** for the **analysis of the cells** in the **2.5D model(s)**?

- Multiple choice
- Options:
  - I do not use 2.5D models
  - Cell viability assays
  - Optical microscopy
  - Electron microscopy
  - Flow cytometry
  - Immunoblot
  - qRT-PCR
  - Cryosectioning
  - Oxygen measurement
  - Other (specify)

c. What technique(s) do you **use** for the **analysis of the cells** in the **3D model(s)**?

- Multiple choice
- Options:
  - I do not use 3D models
  - Cell viability assays
  - Optical microscopy
  - Electron microscopy
  - Flow cytometry
  - Immunoblot
  - qRT-PCR
  - Cryosectioning
  - Oxygen measurement
  - Other (specify)

d. What technique(s) do you **use** for the **analysis of the cells** in the **other model(s)**?

- Multiple choice
- Options:
  - I do not use 2D models
  - Cell viability assays
  - Optical microscopy
  - Electron microscopy
  - Flow cytometry
  - Immunoblot
  - qRT-PCR
  - Cryosectioning
  - Oxygen measurement
  - Other (specify)

30. What are the **experimental outputs / downstream applications** for your *in vitro* culture assays? * Please, select what type of *in vitro model* you use for the following experiments:

- Multiple choice questions

a. Proliferation/Migration/Invasion assays

- Options:

– I do not do proliferation/migration/invasion assays
- 2D Culture
- 2D Co-Culture
- 2.5D Culture
- 2.5D Co-culture
- 3D natural scaffold
- 3D natural scaffold Co-Culture
- 3D synthetic scaffold
- 3D synthetic scaffold Co-Culture
- 3D semi-synthetic scaffold
- 3D semi-synthetic scaffold Co-Culture
- 3D scaffold-free culture
- 3D scaffold-free culture Co-Culture
- Microfluidic cell culture platforms / organ-on-a-chip
- Hybrid System

b. Drug screening assays

- Options:

- I do not do drug screening assays
- 2D Culture
- 2D Co-Culture
- 2.5D Culture
- 2.5D Co-culture
- 3D natural scaffold
- 3D natural scaffold Co-Culture
- 3D synthetic scaffold
- 3D synthetic scaffold Co-Culture
- 3D semi-synthetic scaffold
- 3D semi-synthetic scaffold Co-Culture
- 3D scaffold-free culture
- 3D scaffold-free culture Co-Culture
- Microfluidic cell culture platforms / organ-on-a-chip
- Hybrid System

c. Angiogenesis assays

- Options:

- I do not do angiogenesis assays
- 2D Culture

- 2D Co-Culture
- 2.5D Culture
- 2.5D Co-culture
- 3D natural scaffold
- 3D natural scaffold Co-Culture
- 3D synthetic scaffold
- 3D synthetic scaffold Co-Culture
- 3D semi-synthetic scaffold
- 3D semi-synthetic scaffold Co-Culture
- 3D scaffold-free culture
- 3D scaffold-free culture Co-Culture
- Microfluidic cell culture platforms / organ-on-a-chip
- Hybrid System

d. Cellular uptake & cellular release assays

▪ Options:

- I do not do cellular uptake & cellular release assays
- 2D Culture
- 2D Co-Culture
- 2.5D Culture
- 2.5D Co-culture
- 3D natural scaffold
- 3D natural scaffold Co-Culture
- 3D synthetic scaffold
- 3D synthetic scaffold Co-Culture
- 3D semi-synthetic scaffold
- 3D semi-synthetic scaffold Co-Culture
- 3D scaffold-free culture
- 3D scaffold-free culture Co-Culture
- Microfluidic cell culture platforms / organ-on-a-chip
- Hybrid System

e. Immune cell response assays

▪ Options:

- I do not do immune cell response assays
- 2D Culture
- 2D Co-Culture
- 2.5D Culture
- 2.5D Co-culture
- 3D natural scaffold
- 3D natural scaffold Co-Culture
- 3D synthetic scaffold
- 3D synthetic scaffold Co-Culture
- 3D semi-synthetic scaffold

- 3D semi-synthetic scaffold Co-Culture
- 3D scaffold-free culture
- 3D scaffold-free culture Co-Culture
- Microfluidic cell culture platforms / organ-on-a-chip
- Hybrid System

f. Extracellular vesicles' *in vitro* function assays

▪ Options:
- I do not do extracellular vesicle assays
- 2D Culture
- 2D Co-Culture
- 2.5D Culture
- 2.5D Co-culture
- 3D natural scaffold
- 3D natural scaffold Co-Culture
- 3D synthetic scaffold
- 3D synthetic scaffold Co-Culture
- 3D semi-synthetic scaffold
- 3D semi-synthetic scaffold Co-Culture
- 3D scaffold-free culture
- 3D scaffold-free culture Co-Culture
- Microfluidic cell culture platforms / organ-on-a-chip
- Hybrid System

g. Gene manipulation assays

▪ Options:
- I do not do gene manipulation assays
- 2D Culture
- 2D Co-Culture
- 2.5D Culture
- 2.5D Co-culture
- 3D natural scaffold
- 3D natural scaffold Co-Culture
- 3D synthetic scaffold
- 3D synthetic scaffold Co-Culture
- 3D semi-synthetic scaffold
- 3D semi-synthetic scaffold Co-Culture
- 3D scaffold-free culture
- 3D scaffold-free culture Co-Culture
- Microfluidic cell culture platforms / organ-on-a-chip
- Hybrid System

h. Other: Please specify the application and what type of *in vitro* model you use **(not mandatory)**

31. Have you published on any kind of novel *in vitro* model?

- Yes → **Go to Question 32**
- No → **Go to Question 33**

32. Optional: Please, include DOI/PMID for the publications.

- Comment

33. Do you perform additional *in vivo* studies in your laboratory?

- Yes → **Go to Question 34**
- No → **Go to Question 36**

34. If you use in vivo models, please indicate which *in vivo* model you use

- Comment → **After this question, go to question 35**

35. Is your research based predominantly *on in vitro* or *in vivo* studies? → **After this question, Go to Question 37**

- *In vitro*
- *In vivo*

36. If you do not use *in vivo* models, what are the main reasons for that?

- Multiple choice question
- Options:
  - Lack of availability
  - The 3Rs (replacement, reduction, refinement)
  - Lack of resources
  - Personal choice
  - Other: (specify)

37. How much do you agree or disagree with the following statements?

- Rating scale question
- Affirmations:
  - If both techniques (*in vitro*/ *in vivo*) could achieve the same result, I prefer to use an *in vitro* approach.
  - I am interested using 3D culture models
  - I am reluctant to invest a lot of time and resources into developing a 3D model culture as I am not confident of its success.
  - I think that it is still not feasible to completely replace *in vivo* with *in vitro* models.
  - 2D cell culture models should be completely replaced by 3D cell culture models.
  - My main concern with the use 3D culture models is that many assays are not adapted for them.

38. From your experience, list the 3 main benefits and 3 main limitations of using 3D *in vitro* culture system

a. Main benefits (Comment box)
b. Main limitations (Comment box)

39. If you would like to suggest how the use of 3D models could be improved to enhance cancer research, please do so below (Comment box)

40. If you would like to add any other comments/suggestions/remarks, please do so below (Comment box)

Thanks Page

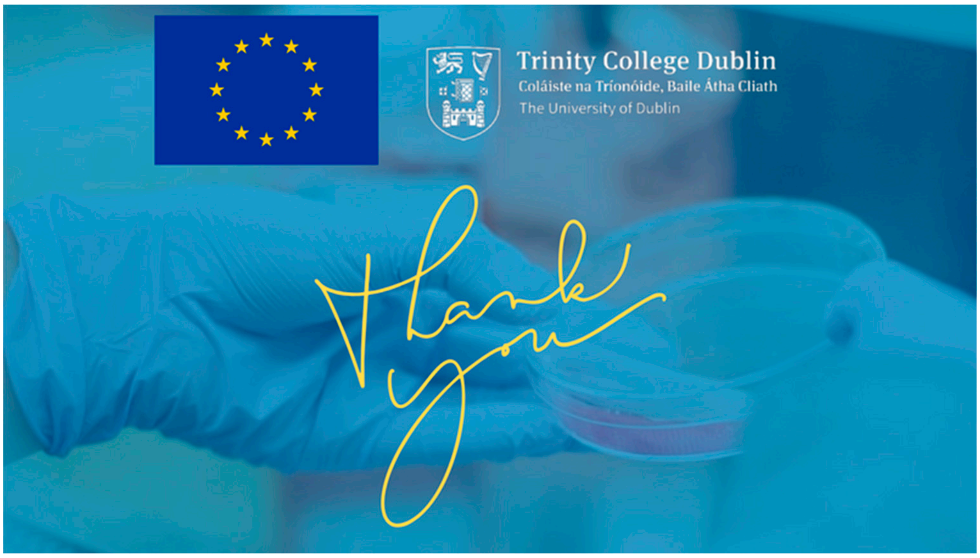

Thank you for your time and effort in completing this survey.

Your input is important to us. When the Survey is completed, we will analyze the information and share the outcome.

Please forward this Survey to anyone who you fell might be interested in participating. You can also share using the social media sharing buttons that appeared below:
